# Annotations

**Published:** 1893-08-26

**Authors:** 


### ANNOTATIONS.

**The Home or the Workhouse?**

We have all heard of the little "slavey," fresh from the workhouse, who did not know how to blow out a candle, having all her life seen only gas, lighted by an official told off for the purpose. Even if not strictly a matter of fact, the story indicates the ignorance of common things, common duties, common responsibilities, which marks those who have been brought up in "institutions." They have had so much of their thinking done for them all their lives that they are practically unfit to take their part in the work of the world. In some wisely-governed unions, however, the guardians have adopted the plan of boarding out the pauper children in cottage homes. Miss Florence Davenport Hill, who is a thoughtful and energetic member of the Hampstead Board of Guardians, writes on this subject in a recent number of the *Economic Journal*. She points out that this mode is, even in the vulgar sense of the word, economical. The annual cost of boarding out a child varies from £10 to, at the outside, £15, while keeping them in the workhouse means an expenditure of £18, £20, or more. Of course, it is necessary to choose suitable homes, not to plant the children in mere baby farms; but with careful supervision on the part of guardians—and here the lady guardian can render valuable service—it is not difficult to find proper places. A childless couple, or a pair of elderly folks whose own nestlings have gone out into the world, will welcome the little stranger, make it indeed their own, until the very name of pauper is forgotten. The boarder shares the work of the house, becomes interested in its duties, begins to talk of the foster parents' house or garden as "ours"—in short, becomes a member of a family. This family life is the best training for family life, in which the best hopes of the future lie. It has been proved in many instances that these boarded-out children do well in after life. Comparatively few of their children become paupers in turn, while generation after generation of those brought up in the workhouse come back to their parents' home. Boarding-out means up-bringing on natural lines. As such, it is almost bound to be successful.

**Where are the British?**

In another column we deal with the excessive heat of the climate during the past month. It has been well said that in response to the kisses of her lord, the sun, Nature awakes to loveliness and abundance, and the children of earth are blessed in the open-handed fruitfulness of the glorious summer. Man takes an opposite course. Thus the present management of the House of Commons and its business is tending in the direction of the destruction of the minds and bodies of its members. It is urged as an excuse by one party that every law of nature and health must be set aside in order to force through legislation of a particular kind. It is urged by the other party that resistance to such a policy is vital to the well-being of England. The first party insist that a policy of drive, scramble, and rush will enable them, at an autumn session, to pass measures which the well-being of the people imperatively demands. If there be any force in this contention, it must be remembered that the passage of good laws can only be attained under conditions which will enable the members of the legislature to devote themselves to business with healthy minds and sound bodies. The heat, when faced with the wisdom of tropical experience, has been trying enough to ordinary people, but such heat in an atmosphere of worry, excitement, and party strife, must prevent the enactment of sound laws, and lead to the degradation of the legislature. Britons, we use the term to include residents north and south of the Tweed, used to be famous for their common sense. Has not the time arrived, if this ancient reputation is to be upheld, when, apart from politics and parties altogether, Britons must assert themselves, and combine to conserve the reputation of the nation by insisting that practical common sense shall sway the members of the House of Commons? As physiologists, we know that it will be impossible for the constitution of the individual Parliament man to sustain the strain which young, thoughtless, and interested partisans are endeavouring to subject it to. We make bold to say there is not a level-headed Briton outside the House of Commons who does not regard the present state of things there as verging on lunacy, and who does not feel that if the members of the House of Commons have forgotten what is due to their sires and to themselves, immediate steps must be taken to prevent the destruction of the health of Parliament and its members. No useful laws, no measures which will meet the requirements of the people to the full, and no legislation of the best kind can emanate from an assembly which continuously sits in the present atmosphere, and which declines to take an adequate and most necessary holiday. Britons require sound legislation, not the ill-considered, hurried, and incomplete enactments of excited, over-wrought and worried men. It is time that the sober sense of the British people, in and out of Parliament, should secure that the present atmosphere of insanity which pervades the House of Commons shall be ended or mended with all necessary despatch. Every British member should be speedily made to feel that the commonwealth demands either an adequate holiday for our legislators or the immediate dissolution of the present House of Commons on the simple grounds of its impotence for legislative effort and its insanity of procedure.

**Moral and Physical Analogy.**

A small amount of thought brings out the analogy between moral and physical disease. Our dealings with moral disease are turned rather towards the punishment than the prevention; prevention, that is to say, such as is arrived at by grasping the evil at its birth, or even going further, and seeing that all moral environments shall be healthy. We are inclined to look upon crime as a spontaneous impulse, not as a thing of steady growth; the natural outcome of certain habits and surroundings. We make no allowance, no provision for the training of those tainted by crime through inheritance. Not yet, that is to say, though public opinion is slowly grasping the fact that the inheritance of physical disease is a very real fact, and that those who come of weakly parents require a special building up and care which those who are born of healthy parents do not. The moral law is very much the same. A predisposition to certain crimes is the way the sins of the fathers fall on the children. The crime of the child is met as was the crime of the father, by punishment, which is to act by force of example against all the strong effects of tendency and surrounding. It does not do so. It is not to the hangman that we must look for the diminution of the crime of murder. In those States in America where capital punishment does not exist, murderers are fewer than in those where it obtains. By this we are not arguing for the abolition of punishment, nor for the futile system of excuse, because "they cannot help it." Something else is wanted. The diseased want special care and treatment. Religion has done much, but its exponents, beyond classing all as sinners, have failed, speaking in diagnosis. Physically, if we were suffering from bronchitis, the treatment for typhoid would not avail. We want more moral comprehension. We want to grasp the history of each moral plant, the suitable atmosphere and soil for its growth, and the grafting and pruning that is necessary, and then the crime disease may clear and leave our horizon healthy and sound.

